# Complex Immunometabolic Profiling Reveals the Activation of Cellular Immunity and Biliary Lesions in Patients with Severe COVID-19

**DOI:** 10.3390/jcm9093000

**Published:** 2020-09-17

**Authors:** Adam Klocperk, Marketa Bloomfield, Zuzana Parackova, Irena Zentsova, Petra Vrabcova, Jan Balko, Grigorij Meseznikov, Luis Fernando Casas Mendez, Alzbeta Grandcourtova, Jan Sipek, Martin Tulach, Josef Zamecnik, Tomas Vymazal, Anna Sediva

**Affiliations:** 1 Department of Immunology, 2nd Faculty of Medicine, Charles University and University Hospital in Motol, 150 06 Prague, Czech Republic; marketa.bloomfield@ftn.cz (M.B.); zuzana.parackova@fnmotol.cz (Z.P.); irena.zentsova@fnmotol.cz (I.Z.); petra.vrabcova@lfmotol.cuni.cz (P.V.); anna.sediva@fnmotol.cz (A.S.); 2 Department of Pediatrics, 1st Faculty of Medicine, Charles University and Thomayer’s Hospital, 140 59 Prague, Czech Republic; 3 Department of Pathology and Molecular Medicine, 2nd Faculty of Medicine, Charles University and University Hospital in Motol, 150 06 Prague, Czech Republic; jan.balko@fnmotol.cz (J.B.); josef.zamecnik@lfmotol.cuni.cz (J.Z.); 4 Department of Infectious Diseases, University Hospital in Motol, 150 06 Prague, Czech Republic; grigorij.meseznikov@fnmotol.cz (G.M.); martin.tulach@fnmotol.cz (M.T.); 5 Department of Pneumology, 2nd Faculty of Medicine, Charles University and University Hospital in Motol, 150 06 Prague, Czech Republic; Luis.Mendez@fnmotol.cz (L.F.C.M.); alzbeta.grandcourtova@fnmotol.cz (A.G.); 6 Department of Anesthesiology and Intensive Care Medicine, 2nd Faculty of Medicine, Charles University and University Hospital in Motol, 150 06 Prague, Czech Republic; jan.sipek@fnmotol.cz (J.S.); tomas.vymazal@fnmotol.cz (T.V.)

**Keywords:** COVID-19, SARS-CoV-2, immunity, T cells, IL-6, GGT, hepatopathy, biliary lesion, acute kidney injury, liver disease

## Abstract

This study aimed to assess the key laboratory features displayed by coronavirus disease 2019 (COVID-19) inpatients that are associated with mild, moderate, severe, and fatal courses of the disease, and through a longitudinal follow-up, to understand the dynamics of the COVID-19 pathophysiology. All severe acute respiratory syndrome coronavirus 2 (SARS-CoV-2)-positive patients admitted to the University Hospital in Motol between March and June 2020 were included in this study. A severe course of COVID-19 was associated with an elevation of proinflammatory markers; an efflux of immature granulocytes into peripheral blood; the activation of CD8 T cells, which infiltrated the lungs; transient liver disease. In particular, the elevation of serum gamma-glutamyl transferase (GGT) and histological signs of cholestasis were highly specific for patients with a severe form of the disease. In contrast, patients with a fatal course of COVID-19 failed to upregulate markers of inflammation, showed discoordination of the immune response, and progressed toward acute kidney failure. COVID-19 is a disease with a multi-organ affinity that is characterized by the activation of innate and cellular adaptive immunity. Biliary lesions with an elevation of GGT and the organ infiltration of interleukin 6 (IL-6)-producing cells are the defining characteristics for patients with the fulminant disease.

## 1. Introduction

Severe acute respiratory syndrome coronavirus 2 (SARS-CoV-2) is a novel human coronavirus that has caused a swiftly spreading disease named COVID-19, which was defined as a pandemic by the World Health Organization in February 2020 [1,2]. Millions have been infected worldwide and hundreds of thousands have died, with the global estimated totals changing rapidly over time. Most people (about 80%) who acquire COVID-19 experience mild to moderate symptoms and recover without special treatment [3]. However, a subgroup of patients develops a severe form of the disease with a high mortality rate, which is hallmarked by severe respiratory distress syndrome, sepsis, coagulation disorder, or even multiple organ failure [4,5,6,7].

Although the exact pathogenesis of the virus-induced damage is not yet known, several mechanisms have been proposed. The surface spike protein of SARS-CoV-2 binds to the angiotensin-converting enzyme-2 (ACE2) receptors [8,9,10] expressed in the alveolar epithelia of the lungs, kidneys, hepatocytes, epithelial cells of the bile ducts, the vascular endothelium, and other cells [11,12,13,14]. Other potential SARS-CoV-2 receptors, such as CD147 or CD26, have also been identified and are expressed in immune cells [15]. Accordingly, the SARS-CoV-2 organotropism extends beyond the respiratory tract [16]. Endothelitis, alveolar damage, and thrombotic microangiopathy have been described in the lungs and kidneys, which is accompanied by the infiltration of mononuclear cells and macrophages [12,17,18].

An efficient, well-coordinated host immune response is a crucial first-line antiviral defense. In severe COVID-19 patients, several studies have documented various degrees of immune dysregulation that affect both innate and adaptive immunity, which may result in immune-mediated tissue injury [19,20]. The recruitment and activation of immune cells, particularly neutrophils, is accompanied by an exuberant release of pro-inflammatory cytokines and chemokines—a so-called “cytokine storm” [4,21,22]. Along with a simultaneous decrease in the monocytes, eosinophils, and basophils [20,23], marked lymphopenia and the functional exhaustion of CD8 T cells and natural killer (NK) cells have been associated with a severe course of the disease [24,25,26,27]. 

Various prognostic markers for the increased severity and mortality in adult COVID-19 disease have been proposed in several heterogeneous studies, including male sex; older age; pre-existing lung, cardiac, renal, and liver disease; hypertension; obesity [28,29,30,31]. Individually, laboratory abnormalities have been reported in COVID-19 patients, including an elevation of inflammatory markers and liver enzymes, abnormal renal function tests, and an elevated serum soluble interleukin 2 (IL-2) receptor (sIL2R) and IL-6. Coagulopathy associated with elevated D-dimers has also been frequently observed among severe COVID-19 patients [4,32]. 

The current clinical knowledge pool for research on COVID-19 disease relies on largely heterogeneous cohort studies of various scales and individual objectives. Therefore, we chose to prospectively follow all patients with a verified SARS-CoV-2 infection admitted to our hospital and construct a rich dataset derived from a single-center cohort of patients that was stratified based on disease severity. The dataset also featured key clinical information and a complex high-parametric laboratory profile of all patients spanning metabolic, hematologic, and immune parameters. The cohort was followed longitudinally throughout the disease. The studied parameters were selected based on previously published COVID-19 data and the best local clinical practice, spanning both features important for disease pathogenesis and markers helpful for the clinical management of the patients.

## 2. Patients and Methods

### 2.1. Patient Cohort and Study Design

All patients admitted to the University Hospital in Motol, Prague, Czech Republic, between March and May 2020 who tested positive for the presence of SARS-CoV-2 RNA in a nasopharyngeal swab using a reverse real-time polymerase chain reaction (rtPCR) were included in this study. Patients were retrospectively divided into subcohorts based on the severity of the disease course as follows: patients with a moderate course of the disease had clinical signs of pneumonia (cough and auscultation) and verified infiltration on a chest X-ray or computed tomography; patients with a severe course of the disease required mechanical ventilation; patients with a mild course of the disease did not fulfill any of the above criteria, but had a positive SARS-CoV-2 nasopharyngeal swab rtPCR; patients with a fatal course of the disease died during the study. Patients included in the severe cohort were only included in the study if they exhibited a stable remission of symptoms allowing for their transfer from the intensive care unit. A summary of the overall cohort, including the cohort size, age, and sex, as well as the basic clinical characteristics of each subcohort, is given in Table 1.

This study was carried out following the recommendations of the Ethical Committee of the Second Faculty of Medicine, Charles University in Prague and the University Hospital in Motol, Czech Republic. The protocol was approved by the Ethical Committee. All subjects gave written informed consent following the Declaration of Helsinki.

### 2.2. Laboratory Parameters

Routine in-house methods were used for an evaluation of all laboratory parameters included in this study. Details concerning individual laboratory methods are available from the authors upon request.

For an evaluation of the serum anti-SARS-CoV-2 antibodies, the EDI™ Novel Coronavirus COVID-19 immunoglobulin M (IgM) or IgG ELISA Kits (EDI Epitope Diagnostics, Inc., San Diego, CA, USA) were used and the data were acquired using a QUANTA-Lyser 3000 (Inova Diagnostics, San Diego, CA, USA).

### 2.3. Lymphocyte Subsets

Lymphocyte subsets were evaluated using flow cytometry. Full blood was drawn into ethylenediaminetetraacetic acid (EDTA)-coated tubes and then stained according to the manufacturer’s instructions using the DryFlowEx ASC Screen Kit, the DryFlowEx ACT T Screen Kit, and the EXCELLYSE I lysing kit (all from EXBIO, Prague, Czech Republic). Data were acquired on a BD LSR II Fortessa (BD Biosciences, Franklin Lakes, NJ, USA) and analyzed using FlowJo software (version 10; TreeStar, Ashland, OR, USA).

### 2.4. Immunohistochemistry

Tissue samples were fixed in neutral buffered 4% formaldehyde and embedded in paraffin. For the immunohistochemistry, 3 μm thin histological sections were used. An anti-CD8 antibody (clone C8/144B, Agilent, Santa Clara, CA, USA, dilution 1:200, pre-treatment: heating in a buffer at pH9 in a water bath) and anti-IL-6 antibody (monoclonal antibody against recombinant full-length protein corresponding to Human IL-6 aa 29–212, the clone was not specified by the antibody producer, Abcam, Cambridge, UK, dilution 1:2000, pre-treatment: heating in a buffer at pH6 in a water bath) were employed. Detection was performed using a one-step micropolymeric non-Biotin system (Bio SB, Santa Barbara, CA, USA) with a peroxidase complex and 3,3′-diaminobenzidine tetra-hydrochloride (DAB). The nuclei were counterstained with hematoxylin.

A sample from lung transplantation donor lungs was used as a healthy control for the lung necropsy. A sample from a healthy liver biopsy was used as a healthy control for the liver necropsy.

### 2.5. Statistical Analysis

In the boxplots used throughout the manuscript, boxes depict the 25th and 75th percentiles (first and third quartile, respectively) and whiskers depict the 2.5–97.5th percentiles. Student’s *t*-tests with Holm’s multiple comparison adjustment were used for an assessment of the differences between groups. In the correlation graphs in Figure 4 and the correlation matrices in Figure 5, Spearman’s correlation was used.

Statistical analyses and the generation of graphs were performed in the statistical language and environment R, version 3.6.3, using the “ggplot2,” “ggpubr,” and “corrplot” packages; GraphPad Prism software (version 8.0.1; GraphPad Software, San Diego, CA, USA); Microsoft Excel 2016 (Microsoft, Redmond, WA, USA). The Simplified Presentation of Incredibly Complex Evaluations (SPICE) plots shown in Figure 2 were constructed using the SPICE 6 software [33].

## 3. Results

### 3.1. Clinical Course of the Disease

Of the 37 patients included in this study, 10 (27%) had a mild course of COVID-19 characterized by a few clinical symptoms, particularly a fever, myalgia, arthralgia, or a general malaise. Patients with a moderate course of the disease (*n* = 13, 35%) were chiefly characterized by a cough, dyspnea, and the necessity of oxygen therapy; however, they did not require mechanical ventilation. Patients who suffered from a severe course of the disease (*n* = 7, 19%) required admission to an intensive care unit and mechanical ventilation, and in several cases, developed systemic inflammation with multi-organ failure. Finally, seven patients (19%) suffered from a fatal course of the disease after an average of 6.8 ± 10.4 days (mean ± SD) following their admission to the hospital.

The specific characteristics of the cohort, found in Table 1, show that the trend of a severe course of the disease mostly occurred in the elderly, while the younger patients experienced predominantly mild symptoms.

### 3.2. Inflammation

**Figure 1:**
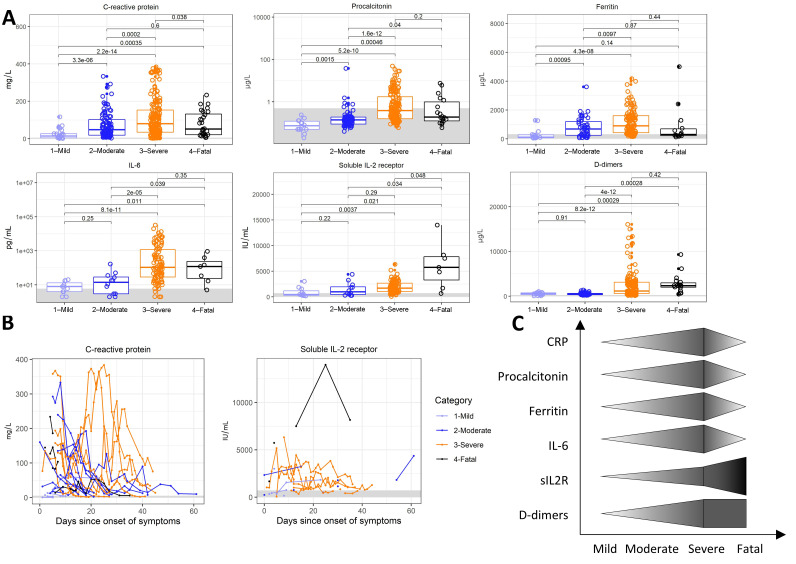
Inflammation. (**A**) C-reactive protein (CRP), procalcitonin, ferritin, interleukin 6 (IL-6), soluble IL-2 receptor (sIL2R), and D-dimers in patients with mild, moderate, severe, and fatal courses of the disease. (**B**) Temporal changes in C-reactive protein and soluble IL-2 receptor levels in patients with mild, moderate, severe, and fatal courses of the disease over time. (**C**) Comparison of the trends between individual parameters shown in (**A**). Boxes depict the median and first and third quartiles, and whiskers show the 2.5th and 97.5th percentiles. Each symbol represents a unique measurement. Multiple measurements at different time points are included for all patients. Values shown are the Student’s *t*-test *p*-values with Holm’s multiple comparison adjustments. Where available, a healthy reference range for adult males is shown in light gray.

While patients with a mild course of the disease only rarely showed an overt elevation of inflammatory markers, such as the C-reactive protein (CRP), procalcitonin, or ferritin (Figure 1A), CRP and ferritin were markedly elevated in the moderate, severe, and fatal subcohorts. High serum IL-6 levels reaching thousands of picograms per milliliter and high procalcitonin were characteristic of severe patients who required mechanical ventilation and had multi-organ involvement.

Interestingly, patients with a fatal course of COVID-19 failed to display an inflammatory response at similarly high levels, which may have contributed to their eventual demise; however, they averaged exceedingly elevated sIL2R levels.

CRP was the highest at the beginning of the disease and decreased rapidly in the first 20 days from the onset of symptoms (Figure 1B); it flared up with multi-organ involvement in the delayed phase of severely ill patients. In contrast, sIL2R remained mostly constant throughout the disease, regardless of the intermittent elevations in CRP levels.

Therefore, we observed a gradual increase of CRP, procalcitonin, ferritin, and serum IL-6 corresponding to the severity of the disease; however, these markers displayed a relative failure to upregulate in patients with a fatal course, who instead displayed high sIL2R and D-dimers (Figure 1C).

### 3.3. Hepatopathy

**Figure 2:**
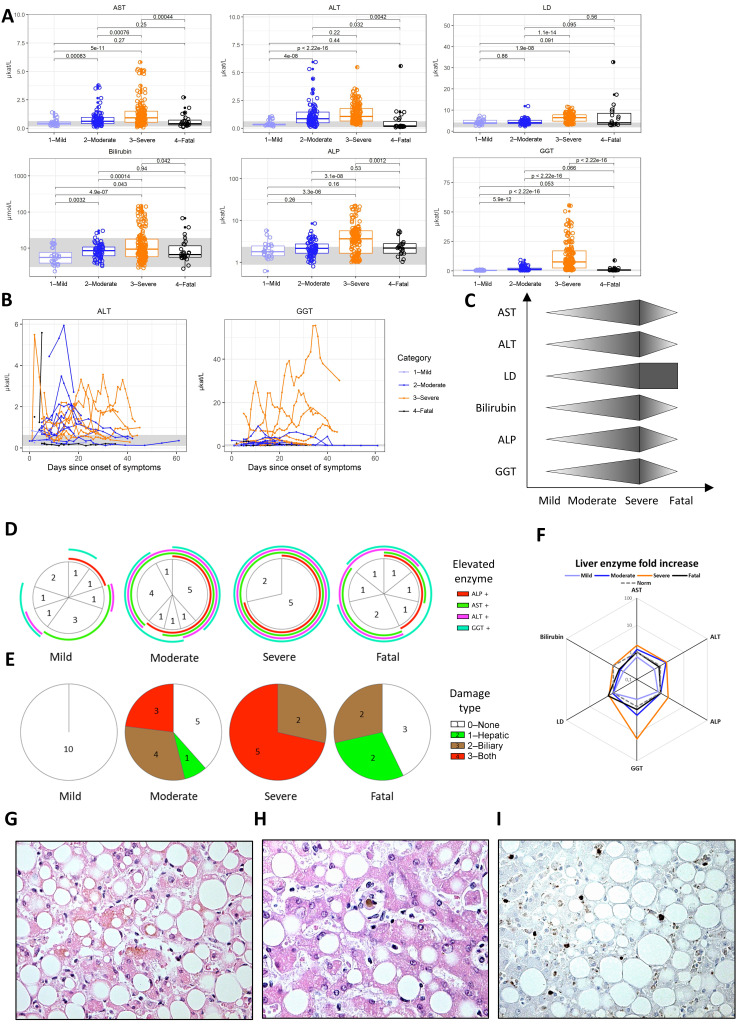
Hepatopathy. (**A**) Aspartate transaminase (AST), alanine transaminase (ALT), lactate dehydrogenase (LD), bilirubin, alkaline phosphatase (ALP), and gamma-glutamyl transferase (GGT) levels in patients with mild, moderate, severe, and fatal courses of the disease. (**B**) Temporal changes in the ALT and GGT levels in patients with mild, moderate, severe, and fatal courses of the disease over time. (**C**) Comparison of the trends among the individual parameters shown in (**A**). (**D**) Elevated enzymes (above a healthy age- and sex-matched reference range) encountered in each subcohort throughout the disease, showing the number of patients with each combination of elevated enzymes. (**E**) Hepatic damage (defined as AST or ALT > 3× the healthy age- and sex-matched reference) and/or biliary damage (defined as GGT or ALP > 2× the healthy reference) encountered in each subcohort throughout the disease. (**F**) Fold increase in each marker of a biliary or hepatic lesion compared to the upper limit of a healthy age- and sex-matched reference range. (**G**) Hematoxilin and eosin staining of a COVID-19 patient liver necropsy (40× magnification) revealing marked macrovesicular steatosis and intracellular cholestasis of hepatocytes. (**H**) Hematoxilin and eosin staining of a COVID-19 patient liver necropsy (40× magnification), which reveals a clot in the biliary tract. (**I**) Immunohistochemical staining of IL-6 in a COVID-19 patient liver necropsy (40× magnification) showing an infiltrate of IL-6-producing cells within sinusoids and the interstitium of the liver parenchyma. Boxes depict the median and first and third quartile, and whiskers show the 2.5th and 97.5th percentiles. Each symbol represents a unique measurement. Multiple measurements at different time points are included for all patients. Values shown are the Student’s *t*-test *p*-values with Holm’s multiple comparison adjustments. Where available, a healthy reference range for adult males is shown in light gray.

The hepatotrophic affinity of SARS-CoV-2 and its hepatopathic qualities have been demonstrated [34]. Correspondingly, we observed an elevation of liver enzymes, i.e., aspartate transaminase (AST), alanine transaminase (ALT), lactate dehydrogenase (LD), or bilirubin, throughout our cohort (Figure 2A). The elevation of ALT peaked at around day 15 from the onset of symptoms and then gradually subsided (Figure 2B).

Additionally, we also registered a significant elevation of gamma-glutamyl transferase (GGT) and alkaline phosphatase (ALP) that was the most pronounced in patients with severe COVID-19 (Figure 2A) and had a more delayed onset than ALT, starting after day 20 on average (Figure 2B). Indeed, while even patients with a mild course of the disease showed some elevation of liver enzymes above a healthy age- and sex-matched reference range (Figure 2D), five out of seven patients with a severe course displayed a consistent elevation of all four enzymes and fulfilled the more stringent laboratory criteria for either a biliary lesion (defined as the elevation of GGT or ALP > 2× the healthy age- and sex-matched reference range values) or both biliary and hepatic (AST or ALT > 3× the reference value) damage (Figure 2E). Of all the enzymes, the elevation of GGT was the most significant and characteristic for severe, but non-fatal COVID-19, with an average of 15 times the healthy age- and sex-matched reference range (Figure 2F).

Cholestasis, which is the collateral feature of biliary injury, was indeed apparent in a liver autopsy from a patient with a fatal course of COVID-19 (Figure 2G), including a clot in the biliary tract (Figure 2H), along with substantial steatosis, despite no previous history of liver disease. Discrete production of IL-6 was detected in the liver (Figure 2I), which was not present in the liver of a non-COVID-19 control (Supplementary Figure S1B), suggesting a role for IL-6 in tissue inflammation and the resulting damage.

### 3.4. Immune Response

The activation of innate immunity is the body’s first-line defense against all types of infectious pathogens, including viruses, although the functional integrity of adaptive immune cells, such as cytotoxic CD8 T cells and NK cells, is the principal component for the final clearance of viral infections.

**Figure 3:**
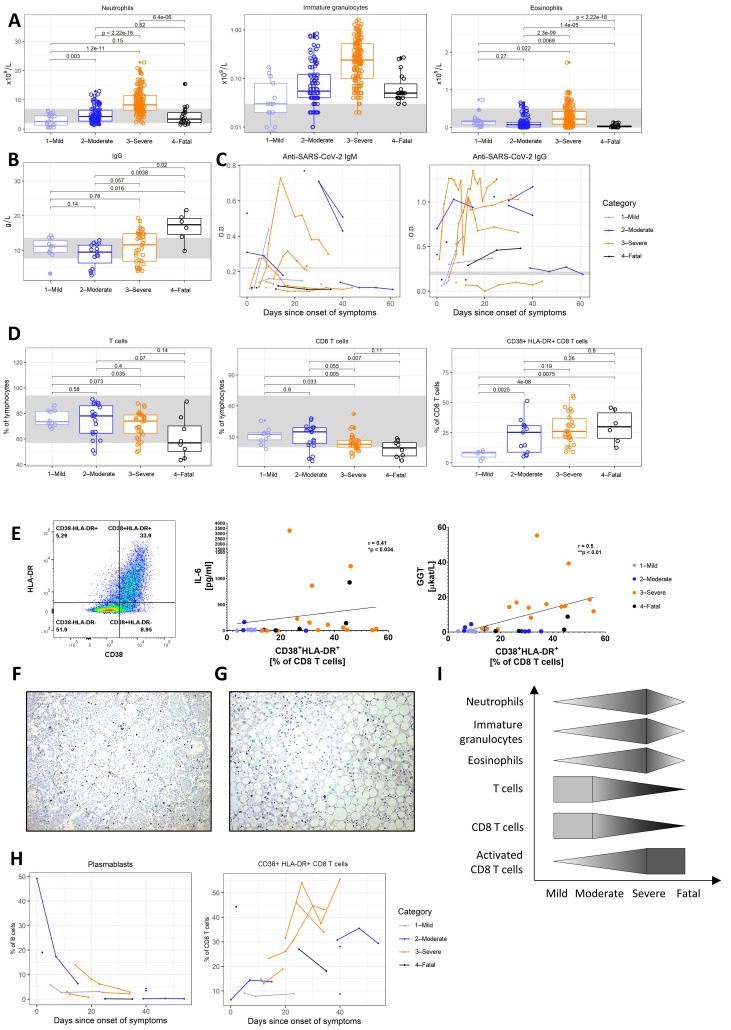
Immunity. (**A**) Neutrophil, immature granulocyte, and eosinophil counts in patients with mild, moderate, severe, and fatal courses of the disease. (**B**) Serum immunoglobulin G (IgG) levels in patients with mild, moderate, severe, and fatal courses of the disease. (**C**) Temporal changes in anti-severe acute respiratory syndrome coronavirus 2 (SARS-CoV-2)-specific IgM and IgG antibodies in patients with mild, moderate, severe, and fatal courses of the disease over time. (**D**) Proportion of T cells, CD8 T cells, and activated (CD38+ HLA-DR+) CD8 T cells in patients with mild, moderate, severe, and fatal courses of the disease. (**E**) Flow cytometry gating strategy for activated CD38+ HLA-DR+ CD8 T cells and their correlation with serum IL-6 and GGT levels, with linear regression trendlines shown and Spearman correlation *r* and *p*-values. (**F**) Immunohistochemical staining of CD8+ cells in a COVID-19 patient lung necropsy (4× magnification) showing cytotoxic T cells within the interstitium and capillaries of interalveolar septa, as well as within alveolar spaces. (**G**) Immunohistochemical staining of CD8+ cells in a COVID-19 patient liver necropsy (20× magnification) showing a lack of CD8 positive cells. (**H**) Dynamics of plasmablast and activated CD38+ HLA-DR+ CD8 T cell populations in patients with mild, moderate, severe, and fatal courses of the disease over time. (**I**) Comparison of trends between the individual parameters shown in (**A**). Boxes depict the median and first and third quartiles, and whiskers show the 2.5th and 97.5th percentiles. Each symbol represents a unique measurement. Multiple measurements at different time points are included for all patients. Values shown are the Student’s *t*-test *p*-values with Holm’s multiple comparison adjustments. Where available, a healthy reference range for adult males is shown in light gray.

Similar to previous studies [4], we found a stark elevation of neutrophils in patients with moderate and especially severe courses of the disease, with a significantly elevated proportion of immature granulocytes (Figure 3A). Of note, eosinophils were also elevated in several patients with a severe course of the disease.

We did not note a major difference in total serum IgG levels between the subcohorts (Figure 3B); however, patients with a fatal course of COVID-19 exhibited significant IgG hypergammaglobulinemia.

The temporal development of specific anti-SARS-CoV-2 antibodies was apparent throughout the disease. Specific IgM antibodies appeared in the first 10 (5–15) days from the onset of symptoms and disappeared after day 15 (13–24), although, in some patients, they remained present for over 30 days (Figure 3C). Virtually concurrent IgM and IgG seroconversions were apparent in all patients, where IgG antibodies showed a better persistence and even gradual increase over time.

Lymphopenia is a well-described negative prognostic factor associated with a severe course of COVID-19 [4]. As part of the lymphopenia in our patients, we specifically noted a decrease of T cells and CD8 T cells in patients with severe and fatal disease courses (Figure 3D). These CD8 T cells were highly activated, co-expressing the surface markers CD38 and Human Leukocyte Antigen – DR isotype (HLA-DR), and were significantly correlated with serum IL-6 levels and the marker of biliary damage, namely, GGT (Figure 3E). While numerous CD8 T cells were found to infiltrate the lungs with histologic signs of interstitial pneumonia in one patient who died from respiratory insufficiency, no such infiltration was found in his liver (Figure 3F), despite the cholestasis and steatosis shown in Figure 2G.

Whereas the humoral immune response displayed within the first 20 days from the onset of the disease led to the fast decrease of plasmablasts detected in the peripheral blood (CD45+, CD19+, CD27hi, CD38hi), the activation of CD8 T cells persisted for over 40 days (Figure 3G). The trends of the immune response to COVID-19 are summarized in Figure 3H.

### 3.5. Kidney and Lung Damage

Most markers of inflammation, the immune response, and liver damage presented in patients with a fatal course of COVID-19 so far seem mostly on par with those seen in patients with a moderate form of the disease, suggesting a weaker response to the infection compared to severely ill patients, which resulted in the patients’ deaths.

**Figure 4:**
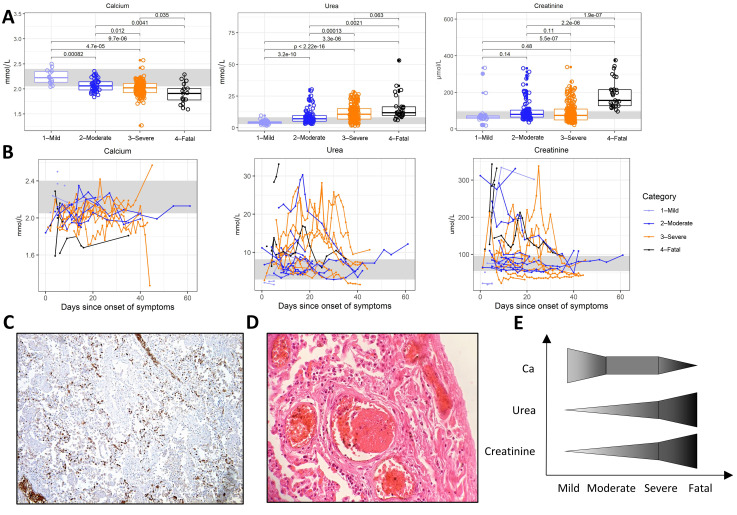
Kidney failure and lung damage. (**A**) Calcium, urea, and creatinine levels in patients with mild, moderate, severe, and fatal courses of the disease. (**B**) Dynamics of calcium, urea, and creatinine levels in patients with mild, moderate, severe, and fatal courses of the disease over time. (**C**) Immunohistochemical staining of IL-6 in a COVID-19 patient lung necropsy (4× magnification) showing IL-6-producing cells within the interstitium and capillaries of interalveolar septa and within the lumen of larger vessels. (**D**) Hematoxylin and eosin staining of a COVID-19 patient lung necropsy (20× magnification) showing interstitial pneumonia and the details of a venous thrombus. (**E**) Comparison of trends between individual parameters shown in (**A**). Boxes depict the median and first and third quartiles, and whiskers show the 2.5th and 97.5th percentiles. Each symbol represents a unique measurement. Multiple measurements at different time points are included for all patients. Values shown are the Student’s *t*-test *p*-values with Holm’s multiple comparison adjustments. Where available, a healthy reference range for adult males is shown in light gray.

Other key characteristics of patients with a fatal course of the disease seen in our study were a mineral disbalance, particularly hypocalcemia, and renal insufficiency, with elevated serum urea and creatinine (Figure 4A). Although elevated urea and creatinine levels were also present in some moderately and severely ill patients, these tended to normalize eventually (Figure 4B).

Although serum IL-6 was not particularly high in fatally ill patients (Figure 1A), there was a substantial production of IL-6 in the lungs, which was driven by interstitially positioned leukocytes (Figure 4C). Pneumonia and acute respiratory distress syndrome were accompanied by numerous thrombi (Figure 4D), along with high plasma D-dimers.

The trends of calcemia and markers of kidney failure in COVID-19 are summarized in Figure 4E.

### 3.6. Discoordination of the Immune Response

As demonstrated above, common trends arose when studying the immune response against the SARS-CoV-2 virus and the different facets of its pathogenicity against humans, as summarized in the trend graphs of Figure 1, Figure 2, Figure 3 and Figure 4.

**Figure 5:**
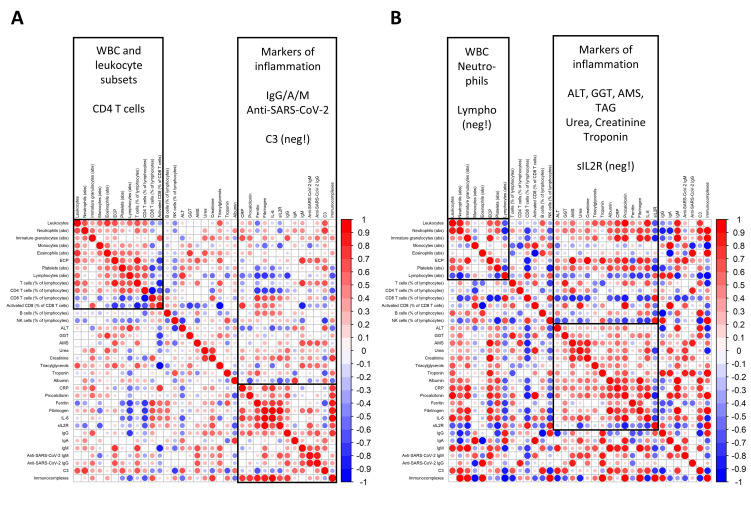
Discoordination of the immune response. (**A**) Heatmap showing the Spearman correlation coefficients between selected parameters mapping the inflammation, immune response, and metabolic parameters in patients with a mild course of the disease. (**B**) Heatmap showing the Spearman correlation coefficients between selected parameters mapping the inflammation, immune response, and metabolic parameters in patients with a fatal course of the disease. The size and opacity of the individual circles represent the Spearman correlation R-value between each pair of variables. A positive correlation is shown in shades of red, and a negative correlation in shades of blue. No sorting algorithm was used; parameters were ordered manually based on thematic groups. WBC: white blood cell, TAG: triacylglycerols

To characterize the complexity of the differences between patients with an efficient, well-coordinated response to the infection, and therefore, only a mild course of the disease, and patients with a fatal course of COVID-19, we constructed correlation matrices of selected laboratory parameters (Figure 5).

In patients with a mild course of COVID-19 (Figure 5A), we found a cluster of positively intercorrelated hematological parameters, such as the overall leukocyte count, neutrophils, and immature neutrophils, but also, interestingly, lymphocytes and T cells. Markers of inflammation, such as CRP, procalcitonin, IL-6, and sIL2R, positively correlated with the humoral immune response—serum IgG, IgA, IgM, and specific anti-SARS-CoV-2 antibodies—clearly showing a well-orchestrated immune response of both the innate and humoral adaptive immunity.

In contrast, patients with fatal COVID-19 (Figure 5B) displayed a negative correlation between leukocytes and lymphocytes, and their inflammatory markers increased with markers of organ failure (liver enzymes, amylase, GGT, urea, and creatinine) and cytotoxic cellular immunity (activated CD38+ HLA-DR+ CD8 T cells) instead. Interestingly, while sIL2R is a marker of inflammation, it showed the opposite trend compared to CRP, procalcitonin, IL-6, or ferritin, which may be driven by its unique elevation in patients with a fatal course of the disease, as seen in Figure 1A.

## 4. Discussion

COVID-19 is a multifaceted disease with a striking stratification of the severity spectrum. As a contribution to the current knowledge pool, our report describes a representative cohort of COVID-19 patients hospitalized during the pandemic in a large Czech hospital. The distribution of mild, moderate, severe, and fatal courses of the disease aligns with previously described cohorts [3,7,22].

Similar to others, we observed a correlation between a set of inflammatory markers, CRP, procalcitonin, ferritin, and serum IL-6, and additionally note that fatal cases failed to mount the corresponding elevation of these parameters, suggesting either the exhaustion or suppression of these key inflammatory components. Instead, patients with a fatal course of the disease showed high sIL2R and D-dimers. Although unspecific, as a marker of T cell activation, sIL2R has been shown to identify patients with multi-organ sarcoidosis [35] in a similar fashion to our patients with fatal COVID-19. The elevation of D-dimers accompanies a hypercoagulation state that manifests as macro- and microvascular thrombotic complications in severe COVID-19 patients [12,17,36], and has been implied as an independent marker of increased mortality [4,32]. Indeed, here we show venous thrombi in the lungs of a deceased COVID-19 patient. Furthermore, by directly demonstrating the presence of IL-6-producing cells and CD8+ T cells in the lungs, we document a cellular inflammation-related mechanism of lung damage beyond the systemic cytokine storm.

Abnormalities in the white blood count, i.e., lymphopenia with marked neutrophilia, are now well-established features of severe COVID-19 that we can confirm in our cohort [20,24]. Additionally, we describe a marked shift toward immature granulocyte forms, which became more pronounced with increasing severity of the disease, and a stark decline in both the mature neutrophil and their precursor counts was found in the fatal courses. The expansion of developing neutrophils in patients with severe COVID-19 was recently identified through single-cell RNA sequencing [37] and their reduction may imply a primary failure to efficiently recruit these innate immune responders.

Moreover, the severe and fatal cases displayed profound T cell, and particularly CD8 T cell, depression, but an unusual presence of activated CD38+ HLA-DR+ CD8 T cells. This reflects the observations that T cells express one of the SARS-CoV-2 receptors CD147 [15], rendering the T cells susceptible to viral entry, and that the infection is associated with a reduction of the naive CD8 T cell percentage [38]. Additionally, activated T cells are more permissive to viral entry and replication [39]. The lymphopenia observed in COVID-19 may, in part, arise as a result of IL-2 signaling inhibition due to the increased soluble IL-2 receptor seen in ours and other cohorts [40]. Taken together, T lymphocyte damage is likely an important aspect of clinical deterioration in COVID-19.

Hepatopathy has been reported in 16–53% of symptomatic patients with COVID-19 [6,41]. Although severe liver dysfunction has been described, the liver injury appears to be mild and transient in the majority of patients, with the median transaminase level remaining lower than twice the upper reference [34,42], which corresponds well with our mild cohort. The elevation of GGT, which is a marker of cholangiocyte injury, has only rarely been reported in COVID-19 so far [34,43]. Interestingly, in our severe, but not fatal, subgroup, we observed an excessive increase of GGT that was strikingly disproportionate to the increase of ALT and AST. The progression to severe disease has previously been associated with predominantly hepatic (elevated ALT and AST) or mixed hepatic and biliary (elevated GGT and ALP) types of liver injury [43]. In our severe cohort, biliary or mixed biliary and hepatic damage was found in the majority of patients. Therefore, we suggest that in COVID-19-related hepatopathy with biliary injury, the predominant elevation of GGT may represent a new independent negative prognostic marker.

Although the hepatopathy and cholestasis present in our cohort of patients may be, at least in part, of hypoxemic or drug-induced origin, the permissiveness of hepatocytes and cholangiocytes to SARS-CoV-2 entry has also been documented [11,44]. Therefore, a direct viral-induced injury to these cells is feasible. To the best of our knowledge, no direct evidence for pro-inflammatory cytokine involvement in the hepatopathy displayed in patients with COVID-19 has been reported. The infiltration of IL-6-producing cells into liver sinusoids and the interstitium may accelerate the production of other markers of inflammation. However, their relative scarcity and the lack of infiltrating CD8 T cells suggests that immune cells, unlike in the lungs, are not the main drivers of pathology in COVID-19 liver disease, despite the correlation between activated CD8 T cells and serum GGT levels.

Overwhelming evidence thus points to the multi-organ affinity of the virus, which also extends to the kidneys [16]. Indeed, our finding of elevated markers of kidney damage in patients with a more severe course of the disease echoes the data from China, where high creatinine and acute kidney injury were risk factors for in-hospital death [45,46]. However, the observed renal pathophysiology is likely multifactorial, involving hypoxemic, hypovolemic, thrombotic, and medication-induced insults.

A comprehensive mapping of markers of the immune and metabolic response in our cohort illustratively documented its uncoordinated orchestration, which was highlighted in the comparison of mild and fatal cases. While systems biology approaches may help to decipher the pathophysiology of COVID-19, especially due to its multi-organ affinity, limitations imposed by heterogeneous cohorts, temporal changes in examined parameters, and interindividual variability due to comorbidities and medication should be kept in mind. These are indeed the main limitations of ours and most other published studies on COVID-19.

## 5. Conclusions

In summary, we demonstrated the complexity of immune and metabolic disturbances in COVID-19 patients. Our experiments contribute to the current understanding of the nature of SARS-CoV-2-driven immunopathology and tissue injury, particularly the systemic inflammation, lymphopenia with T cell activation, and organ infiltration. We observed that severe COVID-19-related hepatopathy may be associated with a marked biliary lesion, which was hallmarked by a stark elevation of GGT, and suggest that this enzyme may represent an additional negative prognostic marker.

## Figures and Tables

**Table 1:** Cohort description.

Cohort	*n* (%) (Sex) ^1^	Age (Years) ^2^	Pneumonia on X-ray	Oxygen Therapy	Mechanical Ventilation	Dialysis	Hydroxychloroquin	Azithromycin	Other Therapy
All patients	37 (100%) (17 F, 20 M)	0.2–96.7 (60.6 ± 27.6)	24 (65%)	21 (57%)	10 (27%)	6 (16%)	15 (43%)	11 (30%)	-
Mild	10 (27%) (6 F, 4 M)	0.2–80.0 (29.8 ± 22.6)	0 (0%)	1 (10%)	0 (0%)	1 (10%)	0 (0%)	0 (0%)	-
Moderate	13 (35%) (6 F, 7 M)	44.4–96.7 (76.8 ± 13.6)	13 (100%)	11 (85%)	0 (0%)	1 (8%)	7 (54%)	4 (31%)	1 steroid
Severe	7 (19%) (3 F, 4 M)	8.5–73.6 (52.0 ± 19.0)	6 (86%)	3 (43%)	7 (100%)	2 (28%)	6 (85%)	4 (57%)	3 steroids 2 tocilizumab 1 remdesivir 1 cytosorb
Fatal	7 (19%) (2 F, 5 M)	56.7–94.8 (83.4 ± 12.5)	5 (71%)	6 (85%)	3 (43%)	2 (28%)	2 (28%)	3 (43%)	2 steroids

^1^ F—female, M—male. ^2^ Age range is shown as the mean ± standard deviation.

## Supplementary Materials

The following are available online at https://www.mdpi.com/2077-0383/9/9/3000/s1. Figure S1: Healthy control biopsy.
