# Supplementary material for: Complex Immunometabolic Profiling Reveals the Activation of Cellular Immunity and Biliary Lesions in Patients with Severe COVID-19
Source: J Clin Med. 2020 Sep 17;9(9):3000. doi: 10.3390/jcm9093000 (PMC7565504; doi:10.3390/jcm9093000)
Supplement: Supplementary file 1 [file jcm-09-03000-s001.pdf]

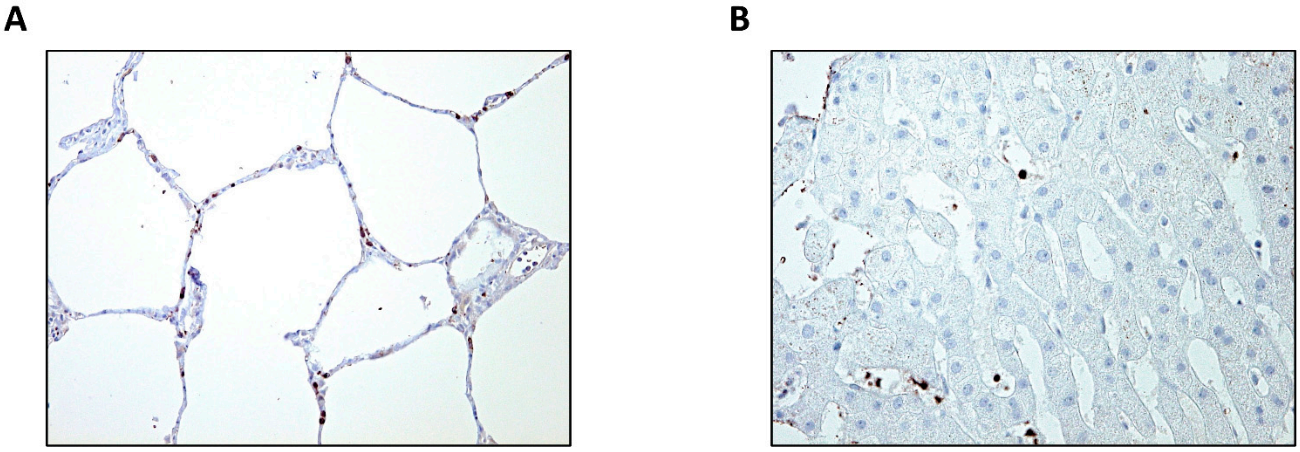

Supplementary Figure 1 Healthy control biopsy

A) Immunohistochemical staining of IL-6 in a healthy lung biopsy (20x magnification).

B) Immunohistochemical staining of IL-6 in healthy liver biopsy (40x magnification) showing sparse IL-6-producing cells within capillaries of liver lobules.
